# Multiplanar knee kinematics-based test battery helpfully guide return-to-sports decision-making after anterior cruciate ligament reconstruction

**DOI:** 10.3389/fbioe.2022.974724

**Published:** 2022-09-16

**Authors:** Lan Zhou, Yihong Xu, Jing Zhang, Luqi Guo, Tianping Zhou, Shaobai Wang, Weidong Xu

**Affiliations:** ^1^ Key Laboratory of Exercise and Health Sciences of Ministry of Education, Shanghai University of Sport, Shanghai, China; ^2^ Department of Orthopedics, Changhai Hospital, The Navy Medical University, Shanghai, China

**Keywords:** return to sport, anterior cruciate ligament reconstruction, kinematics, hop test, isokinetic test

## Abstract

**Background:** There are currently no well-established criteria to guide return to sports (RTS) after anterior cruciate ligament reconstruction (ACLR). In this study, a new test battery consisting of subjective and objective tests, especially multiplanar knee kinematics assessment, was developed to aid RTS decision making after ACLR.

**Methods:** This study was conducted with 30 patients who were assessed a mean of 9.2 ± 0.5 months after ACLR. All patients underwent complete evaluations of both lower limbs with four objective assessments [isokinetic, hop, knee laxity, and 6–degree of freedom (6DOF, angle: flexion-extension, varus-valgus, internal-external rotation; translation: anteroposterior, proximodistal, mediolateral) knee kinematics tests] and two subjective assessments [International Knee Documentation Committee (IKDC) and Anterior Cruciate Ligament Return to Sport after Injury (ACL-RSI) questionnaires]. Limb symmetry indices (LSIs) of knee strength, hop distance, and range of motion (ROM) of knee kinematics were calculated. LSI ≥90%, IKDC scale score within the 15th percentile for healthy adults, and ACL-RSI score >56 were defined as RTS criteria.

**Results:** Significant differences between affected and contralateral knees were observed in the quadriceps strength (*p* < 0.001), hamstring strength (*p* = 0.001), single hop distance (*p* < 0.001), triple hop distance (*p* < 0.001), and rotational ROM (*p* = 0.01). Only four patients fulfilled the overall RTS criteria. The percentages of patients fulfilling individual criteria were: quadriceps strength, 40%; hamstring strength, 40%; single hop distance, 30%; triple hop distance, 36.7%; knee ligament laxity, 80%; flexion-extension, 23.3%; varus-valgus rotation, 20%; internal-external rotation, 66.7%; anteroposterior translation, 20%; proximodistal translation, 33.3%; mediolateral translation, 26.7%; IKDC scale score, 53.3%; and ACL-RSI score, 33.3%.

**Conclusion:** At an average of 9 months after ACLR, objectively and subjectively measured knee functional performance was generally unsatisfactory especially the recovery of knee kinematics, which is an important prerequisite for RTS.

## Introduction

The return to sports (RTS) after anterior cruciate ligament reconstruction (ACLR) has been focus of recent research. However, the timing of safe RTS after ACLR remains unclear. Inappropriate RTS may result in delayed recovery, reinjury and accelerated joint degeneration. A systematic review showed that only 63% of individuals returned to sports at pre-injury levels after ACLR (Webster and Feller, 2019). Among young people, the reinjury rate following postoperative RTS is as high as 35% (Webster and Feller, 2016). Thus, the development of criteria to guide RTS decision making is essential. Consensus has been reached that an extensive test battery including objective physical evaluation is needed for this purpose (Meredith et al., 2020).

Factors influenced RTS after ACLR include, deficits in knee strength and lower-limb neuromuscular control (Barber-Westin and Noyes, 2011; Thomee et al., 2011), psychological factors such as fear of reinjury (Sonesson et al., 2017), and patient-reported outcomes (PROs) (Logerstedt et al., 2014). Based on these risk factors, series of tests and criteria to determine the RTS have been used in recent studies. They include hop tests (Hildebrandt et al., 2015), strength tests (Thomee et al., 2011), use of the jump landing and landing error scoring systems (Gokeler et al., 2017), and psychological readiness assessments (McPherson et al., 2019). At present, the test battery used to evaluate patient RTS after ACLR is still under continuous improvement and on the way to satisfaction.

Recently, in addition to the quantity of movements after ACLR, the quality of movements is of increasing concern when it comes to RTS after ACLR. Several studies have reported persistent kinematic deficits after ACLR, which may cause many adverse effects on patients (Di Stasi et al., 2013; Meredith et al., 2020). A systematic review of 90 studies showed that such knee kinematic asymmetry (e.g., of knee flexion) was a risk factor for post-ACLR reinjury and may contribute to the failure of RTS (van Melick et al., 2016). In addition, biomechanical gait asymmetry after RTS is implicated in the development of osteoarthritis, which has been identified in more than half of patients at 20 years after ACLR (Andriacchi et al., 2004; Cinque et al., 2018). To our knowledge, only a few studies focused on the kinematics of a single plane and no study conducted the assessment of multiplanar knee kinematics when considering the RTS after ACLR currently.

Thus, we developed a new test battery consisted of four objective assessments [isokinetic, hop, knee ligament laxity, and 6–degree of freedom (6DOF, angle: flexion-extension, varus-valgus, internal-external rotation; translation: anteroposterior, proximodistal, mediolateral) kinematics tests] and two subjective assessments [the International Knee Documentation Committee (IKDC) and Anterior Cruciate Ligament Return to Sport after Injury (ACL-RSI) PRO questionnaires]. The limb symmetry index (LSI), by which muscle strength, hop distance, and affected knee kinematics are expressed as percentages of contralateral limb values, is usually used to evaluate knee function recovery after ACLR (Teichtahl et al., 2009; Arhos et al., 2021). In current study, we aimed to use this test battery to identify post-ACLR knee functional deficits after ACLR and explore the importance of multiplanar knee kinematics on RTS decision-making.

## Materials and methods

### Participants

Patients who underwent ACLR in the XXXX hospital and met the inclusion criteria were enrolled in this study. The inclusion criteria were: 1) age 18–40 years; 2) regular participation in sports involving jumping, contact, or pivoting (>50 h/year) (Zarzycki et al., 2018); 3) completeness of required data and voluntary participation; and 4) no previous history of knee injury. The exclusion criteria were: 1) with meniscus repair or multi-ligament reconstruction and 2) development of postoperative complications, including joint fibrosis, pain, effusion, and infection.

**FIGURE 1:**
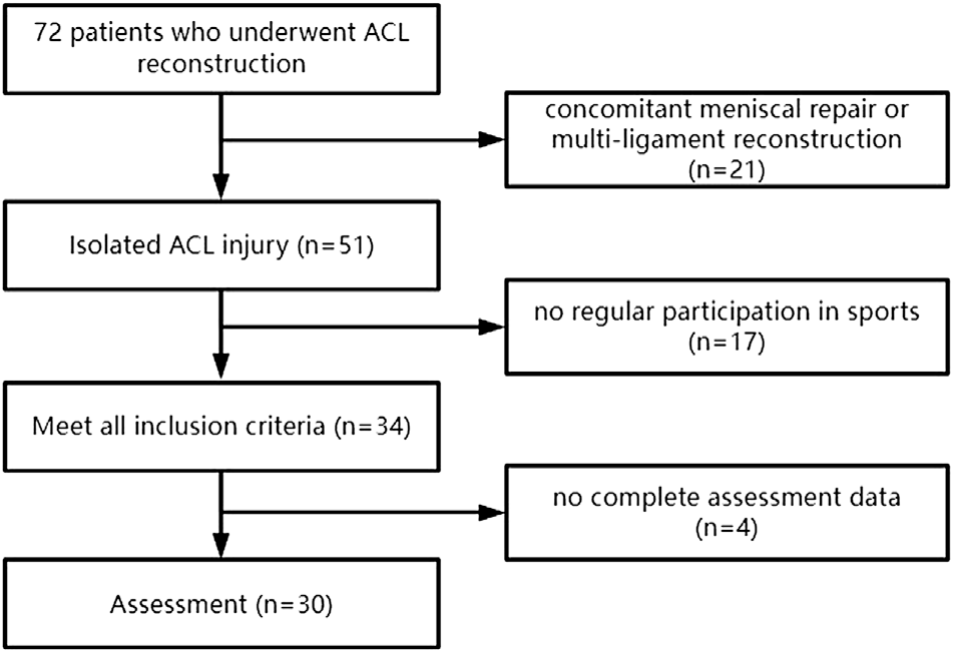
Flow charm of the participants enrollment.

From May 2020 to June 2021, of the 72 patients who underwent ACLR, 21 were excluded due to concomitant meniscal repair or multi-ligament reconstruction, and 17 were excluded because of age or having no regular participation in sports. Of the remaining 34 patients, four were excluded because they did not have complete assessment data. Hence, eventually 30 patients were included in the study (Figure 1). All patients will be clinically examined by our physicians prior to testing. The Institutional Review Board approved the study (NO.102772021RT134) and all patients provided written informed consent.

### Surgical procedure and rehabilitation

An experienced orthopedic surgeon performed all surgeries. During the surgery, the hamstring tendons or synthetic grafts were chosen which were up to the patients to decide. Tunnels were drilled at the tibial and femoral footprints of the ACL using an anteromedial approach. The residual tissues on both sides were preserved. The femoral and tibial ends of the hamstring tendons autografts were fixed with endobuton and intrafix screws, respectively. The range of motion (ROM), graft tension, and possible graft impact were examined under arthroscopy. After surgery, all patients completed a standard postoperative rehabilitation protocol, which focused on the achievement of full knee-extension ROM immediately and knee flexion as tolerated, progression of functional activities, and quadriceps strengthening. Specifically, in the first 2 weeks, patients were encouraged to reduce pain and swelling with ice, elevation, compression and proper rest and passive/active knee flexion and extension to restore knee ROM. In the 3–5 weeks, stairs up and down and neuromuscular training were added. At 12 weeks, patients were allowed to perform running activities and focused on muscle strengthening. All patients were followed up in outpatient clinic 2 weeks, 1 month, 3 months and 6 months after surgery to provide rehabilitation guideline and the test battery was performed 9 months postoperatively. The decision of returning to specific sport was made by the treating surgeon and the physical therapist.

### Subjective RTS assessments

Nine months postoperatively, patients were asked to completed all the assessments. The IKDC scale, considered to be an important measure of successful outcomes after ACLR (Logerstedt et al., 2014), and the ACL-RSI scale were administered to the study participants. The 12-item ACL-RSI scale is used to evaluate the psychological influence (patients’ emotions, confidence in performance, and risk appraisal) of the RTS after ACL (Webster et al., 2008; Sonesson et al., 2017) and has been proven to be a reliable and effective tool for this purpose (Jia et al., 2018). As an RTS criterion, IKDC scale scores within the 15th percentile for normal age- and sex-matched individuals were chosen (Logerstedt et al., 2014). A critical score of 56 points of ACL-RSI scale was used, which could distinguish the difference in psychological readiness between athletes who return to sport after ACLR and those who did not (Ardern et al., 2013).

### Objective RTS assessments

Two experienced rehabilitation therapists conducted the objective assessments (isokinetic, hop, knee laxity, and 6-DOF kinematics tests) in the same laboratory. Quadricep and hamstring strength was measured using an isokinetic dynamometer (Con-Trex^®^ MJ; CMV AG, Dubendorf, Switzerland) and a standard protocol that has been proven to be highly reliable [intraclass correlation coefficient (ICC) > 0.96] (Maffiuletti et al., 2007). All subjects performed three exercises to familiarize them with this task. One set of five isokinetic muscle strength tests was performed at 60°/s. The contralateral knee was assessed first, followed by the affected knee. The mean isokinetic peak torque (Nm) was calculated and normalized to the body weight (Nm/kg). The LSI was calculated as (affected limb value/contralateral limb value) × 100% (Arhos et al., 2021).

Knee laxity was evaluated using a Ligs Digital Arthrometer (Innomotion Inc, Shanghai, China), which was proved with excellent reliability (Chen et al., 2022). The patients placed the knee joint under the direction of the tester. The instrument was then adjusted to increase the forward force slowly from 10 to 133 N. The system recorded the loading force and displacement in real time. ACL injury was suspected when the difference in joint displacement between knees exceeded 3 mm (Arneja and Leith, 2009).

The single-hop (SH) and triple-hop (TH) tests for distance (cm) were performed, in that order, according to the method described by Alexandre et al. (Rambaud et al., 2017). The best distance for each leg from three trials was recorded. The difference between the affected and contralateral limbs was calculated and presented as a percentage (Rambaud et al., 2017). With reference to previous findings (ICC, 0.84–0.92), we considered LSIs >90% for these tests to reliably reflect good recovery (Reid et al., 2007).

The 6DOF knee kinematics test was performed using a marker-based motion analysis system (Opti-Knee^®^, Innomotion Inc, Shanghai, China). The system is based on surgical navigation technology, with an integrated two-head stereo-infrared camera at a frequency of 60 Hz. The total space requirement of the system and test area is about 10 m^2^. During the test, two rigid bodies, each composed of four infrared light reflection markers (OK_Marquer, Innomotion) were connected to each participant’s thigh and shin with bandages. This system was used to obtain a 6-DOF kinematic curve of the tibia relative to the femur in real time during treadmill walking (5 km/h) (Tian et al., 2020), reflecting rotation in three directions [ flexion (+)-extension (-), valgus (+)-varus (-), and external (+)-external (-) rotation] around the *x*, *y*, and *z* axes of the knee’s local coordinate system and displacement [(anterior (+)-posterior (-), lateral (+)-medial (-), and proximal (+)-distal (-)] along these axes. The repeatability of measures obtained with this system has been demonstrated, with tolerances within 1.3 mm translation and 0.9° rotation (Zhang et al., 2015). To assess the symmetry of kinematics between limbs, maximum and minimum values were calculated, and eventually obtained the ROM. To better reflect the knee stability and symmetry, we took the ratio of the smaller value and larger value between two sides of limbs as the LSI. Asymmetry values ≤10% were considered to reflect satisfactory recovery after ACLR (Teichtahl et al., 2009; Arhos et al., 2021).

The RTS test battery is thus used to evaluate fulfillment of the following criteria:

1. IKDC score within the 15th percentile for normal age- and sex-matched subjects (Logerstedt et al., 2014).

2. ACL-RSI score >56 (Ardern et al., 2013).

3. LSI ≥90% peak torque for quadriceps and hamstring strength at 60°/s (Thomee et al., 2011).

4. LSI ≥90% for SH and TH tests (Thomee et al., 2011).

5. Bilateral displacement difference <3 mm (Arneja and Leith, 2009).

6. Difference in ROM between limbs ≤10% in the 6-DOF knee kinematics test (Teichtahl et al., 2009; Arhos et al., 2021)

### Statistical analysis

Continuous and quantitative data are expressed as means ± standard deviations. The Shapiro–Wilk test was used to assess the normality of data distribution. Differences in function between affected and contralateral limbs were analyzed using the paired *t* test or Wilcoxon test, depending on the data distribution. All statistical analyses were conducted using SPSS version 20 (IBM Corporation, Armonk, NY, United States). *p* values <0.05 were considered to be significant.

## Results

### Sample characteristics and subjective assessment outcomes

30 patients (26 men, four women) with a mean age of 27.7 ± 5.9 years were included in the study. The mean postoperative time was 9.2 ± 0.5 months. Detailed information, including the body mass index and graft type, are provided in Table 1. The average IKDC and ACL-RSI scale scores were 81.4 ± 10.6 and 50.5 ± 9.8, respectively.

**TABLE 1:** Participant characteristics.

	Patients
Sex: male/female, n	26/4
Injured knee: left/right, n	12/18
Graft type: HT/SG, n	27/3
Age, y	27.8 ± 5.9
Height, cm	178.7 ± 7.4
Weight, kg	78.2 ± 13.9
BMI, kg/m^2^	24.3 ± 3.4
Time post-surgery, m	9.2 ± 0.5
IKDC score	81.4 ± 10.6
ACL-RSI score	50.5 ± 9.8

HT, hamstring tendon graft; SG, synthetic graft; BMI, body mass index; IKDC, international.

Knee Documentation Committee Subjective Knee Evaluation Form; ACL-RSI, Anterior Cruciate Ligament-Return to Sport After Injury Scale.

### Objective assessment outcomes

Significant differences between affected and contralateral limbs were observed in the quadriceps strength (*p* < 0.001), hamstring strength (*p* = 0.001), SH distance (*p* < 0.001), and TH distance (*p* < 0.001) at 9 months after ACLR. Affected limbs had smaller rotational angles than did contralateral limbs (*p* = 0.01; Table 2); no significant difference in other kinematic parameters was observed. The mean kinematics LSIs did not exceed 80%, except for that for the flexion angle ROM (Table 3).

**TABLE 2:** Functional parameters outcomes^a^.

	Affected limb	Contralateral limb	LSI	*p* Value
Functional tests				
Extensor strength, Nm/kg	1.5 ± 0.5	1.8 ± 0.6	82.9 ± 20.9	<0.001*
Flexor strength, Nm/kg	0.9 ± 0.3	1.1 ± 0.3	85.9 ± 24.1	0.001*
SH, cm	126.2 ± 15.8	145.2 ± 12.7	86.8 ± 6.7	<0.001*
TH, cm	397.3 ± 63.7	460.5 ± 48.2	86.1 ± 8.3	<0.001*
Laxity, mm	16.5 ± 3.0	15.8 ± 3.1	—	0.184

a Data are expressed as mean ± standard deviation; LSI: limb symmetry index; SH: single-hop test; TH: triple-hop test; *Statistically significant difference (*p* < 0.05).

**TABLE 3:** Knee kinematics outcomes.

Knee kinematics	LSI
F-E, °	91.7 ± 6.5
IR-ER, °	77.6 ± 12.4
VR-VL, °	73.2 ± 16.4
A-P, mm	69.7 ± 15.5
P-D, mm	76.0 ± 22.3
M-L, mm	73.8 ± 16.9

LSI, limb symmetry index; F-E, flexion-extension; IR-ER, internal-external rotation.

VR-VL, varus-valgus; A-P, anterior-posterior translation; M-L, medial-lateral translation; P-D, proximal-distal translation.

### Rates of criterion fulfillment

**FIGURE 2:**
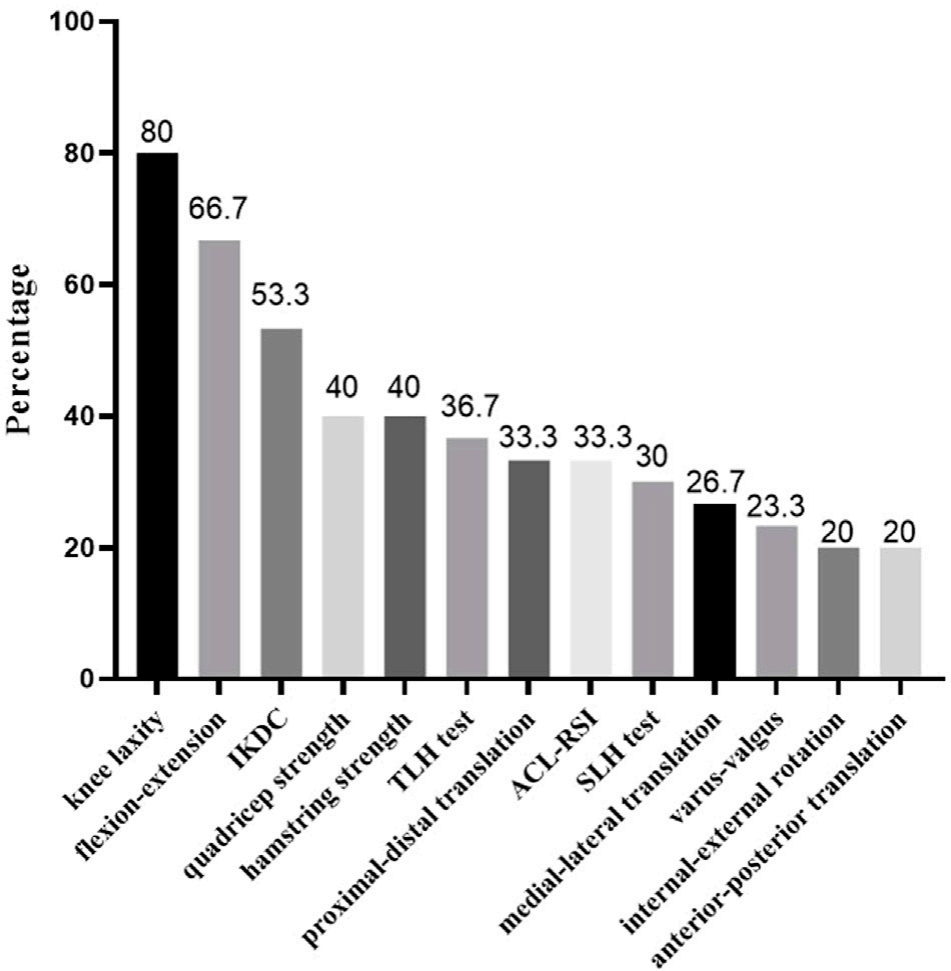
Percentages of participants fulfilling the tests criteria, ranked from high to low.

**FIGURE 3:**
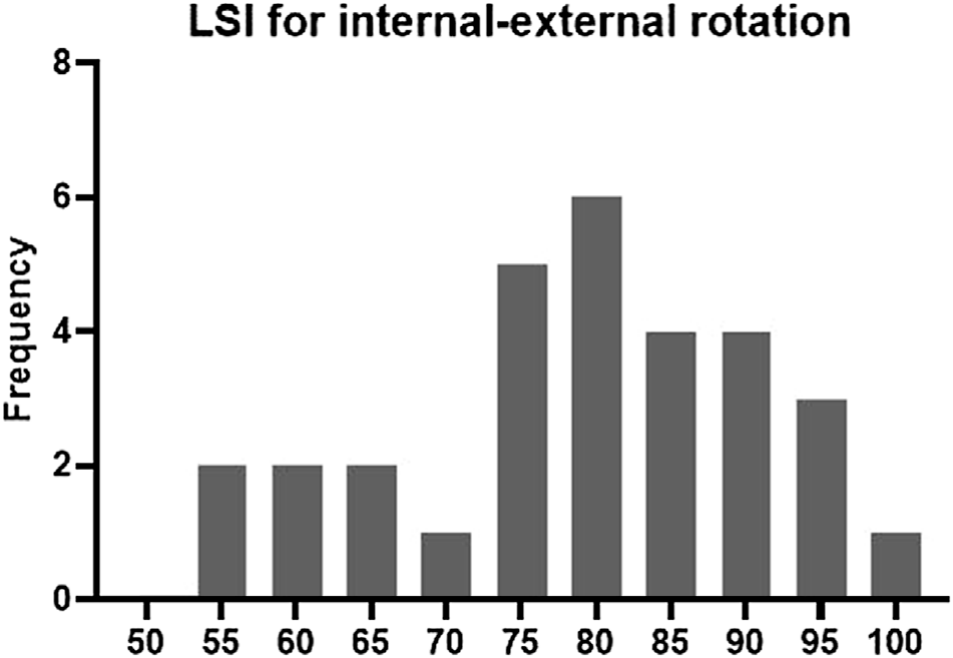
Frequency distribution of internal-external rotation LSIs.

**FIGURE 4:**
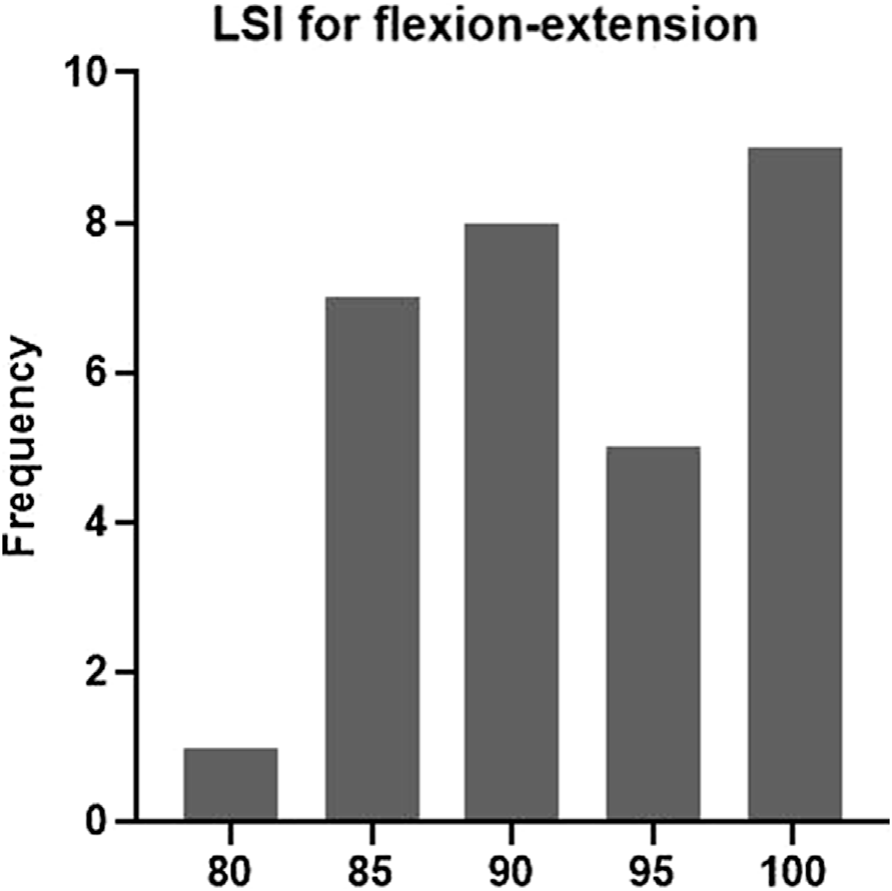
Frequency distribution of flexion-extension angle LSIs.

Four (13.33%) patients fulfilled the criteria of the test protocol. Twelve patients fulfilled the quadriceps and hamstring strength criteria, nine patients fulfilled the SH criterion, 11 patients fulfilled the TH criterion, and 24 patients fulfilled the knee laxity criterion. Twenty, 7, six patients fulfilled the flexion-extension, varus-valgus, and internal-external rotation ROM criterion. Six, 10, and eight patients, respectively, fulfilled the anterior-posterior, proximal-distal, medial-lateral translation ROM criterion. Sixteen patients fulfilled the IKDC scale score criterion and 10 patients fulfilled the ACL-RSI scale score criterion (Figure 2). Representative examples of the frequency distributions of the LSIs for the internal-external rotation and flexion-extension angle are presented in Figure 3 and Figure 4, respectively.

## Discussion

This study found that only 4 (13.3%) of 30 patients fulfilled RTS criteria of the new test battery at approximately 9 months after ACLR, which means further targeted rehabilitation is needed in the future. Notably, only 6 (20%) patients had 6-DOF knee kinematics deficits ≤10%, which implied that knee kinematics may play an important role in indicating the recovery of knee function and RTS.

The low pass rate for the new test battery is similar with previously reported rates for the meeting of strict RTS standards (Thomeé et al., 2012; Gokeler et al., 2017; Raoul et al., 2019). Gokeler et al. (2017) reported that 2 (7.1%) of 28 patients 8 months postoperatively fulfilled all criteria of a test battery consisting of three strength tests, three hop tests, and the Landing Error Scoring System test. Raoul et al. (2019) used a test battery consisting of isokinetic, hop tests and ACL-RSI scale to evaluate the function recovery of patients after ACLR. As a result, only 44 (18.5%) of 234 patients had satisfactory function. The battery developed in this study features the addition of multiplanar kinematic tests. The mean LSI for 6-DOF knee kinematics during running was low, despite good knee laxity and muscle strength symmetry, in our sample, consistent with previous authors’ statements that RTS evaluations after ACLR should focus not only on the quantity, but also the quality, of movement (Kaplan and Witvrouw, 2019; Meredith et al., 2020).

Multiplanar kinematics testing has attracted increasing attention. Asymmetrical kinematics during dynamic movement after ACLR have been observed in several studies. Persistent kinematic abnormalities have been found to be related closely to short-term ACL reinjury after the RTS and long-term osteoarthritis development (Andriacchi et al., 2004; Hewett et al., 2005). Few studies, however, have involved multiplanar kinematics evaluations as part of RTS test batteries. Di Stasi et al. (2013) reported that patients with superior functional outcomes 6 months after ACLR had less asymmetrical gait patterns in the sagittal plane. Similarly, Palmieri-Smith et al. (Palmieri-Smith and Lepley, 2015) observed that patients with less quadriceps strength had greater movement asymmetries in the sagittal plane. In the transverse plane, increased external tibial rotation is related directly to the alteration of the peak contact pressure in the patellofemoral joint (Li et al., 2004). The restoration of knee kinematics after ligament reconstruction may be necessary to protect the knee from abnormal loading (Yoo et al., 2005). In the frontal plane, Mark et al. (Paterno et al., 2010) found that increased valgus deformity during dynamic movement after ACLR is predictor of ACL reinjury after the RTS in athletes. However, kinematic tests have not been successfully implemented routinely in daily clinical practice currently. The test battery used in this study for RTS decision making is clinically practical because it requires little space and takes little time (about 45 min), and is a convenient means of quantifying performance.

RTS decision making based only on the postoperative time is not recommended; and objective measures are considered to be essential (Meredith et al., 2020). Grindem et al. (2016) reported that the post-RTS reinjury rate was 20% higher among patients with quadriceps symmetry indices <90% than among those with indices >90%. Undheim et al. (2015) suggested that isokinetic strength evaluation following ACLR was an important factor when considering an athlete’s readiness for the RTS. Müller et al. (2014) showed that the SH test is one of the strongest predictors of the return to recreational sports activities at 6 months after ACLR. The hop test is generally used to determine the neuromuscular control ability in the knee joint. Abundant evidence shows that such control during sports is poor after ACLR (Hewett et al., 2005). Neuromuscular control is essential to keep the knee joint stable during dynamic activities, and deficiencies may lead to the exertion of greater stress on the passive joint structures (e.g., ligaments and joint capsule), eventually increasing the risk of ACL reinjury. In this study, the mean LSIs for quadriceps strength and SH distance were 82.9 and 86.8%, respectively, but only 40% of patients reached the 90% thresholds for these criteria, reinforcing the importance of LSI thresholds. Ardern et al. (2011) reported patients with SH distance LSIs >85% were more likely to return to pre-injury sports levels. The reason for using 90% LSI in this study was that the participants participated in sports involving jumping, contact, or pivoting regularly before injury and they expected to return to the same type of preinjury sport. It is recommended at least 90% LSI for the affected limb knee when considering RTS (Thomee et al., 2011).

The average IKDC scale score in this study was 81.4, and 15 (50%) patients did not fulfill the criteria recommended for this score by previous researchers (Logerstedt et al., 2014). Low IKDC scale scores can indicate the failure of a series of functional performance RTS tests, including the quadriceps strength and SH tests (Logerstedt et al., 2014). In addition, 20 of 30 patients in this study did not achieve the threshold ACL-RSI scale score of 56 (mean, 50.5). Ardern et al. (2013) found that ACL-RSI scale scores <56 were associated with the failure of RTS. Psychological factors have been shown to influence the RTS after ACLR significantly (Müller et al., 2014; Sonesson et al., 2017; McPherson et al., 2019). Various ACL-RSI scale score cut-offs have been used in different studies, which may be related to differences in subjects’ ages, activity levels, and occupations.

Based on the multiplanar knee kinematics test and other subjective and objective tests results obtained at 9 months after ACLR in this study, suggestions for future rehabilitation and training programs can be offered. For patients, objective test results provide motivation for further rehabilitation, which is very important to reduce the risk of premature RTS. Objective test results may also help enhance patients’ psychological readiness for the RTS.

Functional test remains an important part of RTS consideration after ACLR, and should include the subjective and objective measurement of a series of specific skill. Inadequate RTS tests may not identify residual biological, functional and psychological deficits, which may result in an increased risk of secondary ACL injury when allowing athletes to RTS after ACLR. The ideal test battery content and which tests should be prioritized over others also need to be determined.

Some limitations of the current study should be noted. Firstly, the accuracy of the test battery in the current study in predicting RTS or re-injury after RTS has not been validated. But the primary purpose of this paper was to initiate a new test battery especially adding the multiplanar knee kinematics and highlight the important role of multiplanar knee kinematics in decision-making for RTS after ACLR. Secondly, the participants in this study involved both males and females, and the ligament types included hamstring tendon autograft and synthetic graft, these variables may affect the study results. However, we additionally analyzed the male group and the hamstring tendon autograft group, the trend of the results and the conclusion were consistent with the overall group. In following study, we will continue to include more patients to allow us to perform subgroup analyses. Thirdly, the test battery may be not multifactorial enough to assess safe RTS as it is not clear which components of movement, such as strength, endurance, balance, proprioception are needed individually and in combination to achieve the best effectiveness of safe RTS currently.

## Conclusion

At an average of 9 months after ACLR, only four of 30 patients fulfilled all criteria of the new test battery and only 6 (20%) patients had 6-DOF knee kinematics deficits ≤10%. The knee functional performance especially multiplanar knee kinematics were generally unsatisfactory. Further targeted rehabilitation is needed to release patients to RTS, and knee kinematics may play an important role in indicating the recovery of knee function and RTS.

## Data Availability

The original contributions presented in the study are included in the article/Supplementary Material, further inquiries can be directed to the corresponding authors.
