# Supplementary material for: Multiplanar knee kinematics-based test battery helpfully guide return-to-sports decision-making after anterior cruciate ligament reconstruction
Source: Front Bioeng Biotechnol. 2022 Sep 16;10:974724. doi: 10.3389/fbioe.2022.974724 (PMC9523368; doi:10.3389/fbioe.2022.974724)
Supplement: Supplementary file 1 [file DataSheet1.pdf]

Supplementary material

Analysis for male subjects

Significant differences between affected and contralateral limbs were observed in the quadriceps strength ($P < 0.001$), hamstring strength ($P = 0.002$), SH distance ($P < 0.001$), and TH distance ($P < 0.001$) at 9 months after ACLR. Affected limbs had smaller rotational angles than did contralateral limbs ($P = 0.007$); no significant difference in other kinematic parameters was observed. The mean kinematics LSIs did not exceed 80%, except for that for the flexion angle ROM (Supplementary Table 2).

Three (11.54%) patients fulfilled the criteria of the test protocol. Eleven patients fulfilled the quadriceps and 10 patients fulfilled the hamstring strength criteria, 8 patients fulfilled the SH criterion, 10 patients fulfilled the TH criterion, and 22 patients fulfilled the knee laxity criterion. Eighteen, 6, 5 patients fulfilled the flexion-extension, varus-valgus, and internal-external rotation ROM criterion. Six, 8, and 7 patients, respectively, fulfilled the anterior-posterior, proximal-distal, medial-lateral translation ROM criterion. Fourteen patients fulfilled the IKDC scale score criterion and 8 patients fulfilled the ACL-RSI scale score criterion (Supplementary Table 3).

The trend of the results and conclusion are consistent with the overall group.

Supplementary Table 1 Participant characteristics male subjects

	Patients
male	26
Injured knee: left/right, n	11/15
Graft type: HT/SG, n	25/1
Age, y	27.9±5.9
Height, cm	180.2±6.5
Weight, kg	79.9±12.9
BMI, kg/m^2	24.5±3.0
Time post-surgery, m	9.2±0.5
IKDC score	80.7±11.0
ACL-RSI score	49.5±9.0

HT: hamstring tendon graft; SG: synthetic graft; BMI: body mass index; IKDC: International Knee Documentation Committee Subjective Knee Evaluation Form; ACL-RSI: Anterior Cruciate Ligament-Return to Sport After Injury Scale

Supplementary Table 2 Functional and kinematics parameters [a]

	Affected limb	Contralateral limb	LSI	P value
Functional tests				
Extensor strength, Nm/kg	1.5±0.5	1.8±0.6	84.5±19.8	＜0.001*
Flexor strength, Nm/kg	0.9±0.3	1.1±0.3	85.9±25.2	0.002*
SLH, cm	126.7±15.1	145.2±13.2	87.2±6.0	＜0.001*

TLH, cm	398.8±63.5	460.2±50.5	86.5±7.7	＜0.001*
Knee kinematics				
F-E, °	57.9±5.7	58.1±5.1	90.9±6.4	0.919
IR-ER, °	14.4±3.8	16.8±3.5	76.5±12.4	0.007*
VR-VL, °	12.9±5.0	13.6±4.8	71.1±15.8	0.529
A-P, mm	27.2±9.3	28.6±11.3	69.4±16.0	0.583
P-D, mm	26.0±9.1	24.0±9.8	76.4±22.3	0.294
M-L, mm	15.2±5.0	14.5±6.7	72.8±17.1	0.516

[a] Data are expressed as mean ± standard deviation; F-E: flexion-extension; IR-ER: internal-external rotation; VR-VL: varus-valgus; A-P: anterior-posterior translation; M-L: medial-lateral translation; P-D: proximal-distal translation; *Statistically significant difference (P＜0.05)

Supplementary Table 3 Rates of criterion fulfillment

Pass criteria	Percentage of subjects passed criterion
LSI > 90 % peak torque quadriceps 60°/s	42.3%
LSI > 90 % peak torque hamstrings 60°/s	38.5%
LSI＞90% SLH test	30.8%
LSI＞90% TLH test	38.5%
Bilateral displacement difference of knee joint＜3mm	84.6%
Differences of ROM in flexion-extension＜10%	69.2%
Differences of ROM in varus-valgus＜10%	23.1%
Differences of ROM in internal-external rotation＜10%	19.2%
Differences of ROM in anterior-posterior translation＜10%	23.1%
Differences of ROM in proximal-distal translation＜10%	30.8%
Differences of ROM in medial-lateral translation＜10%	26.9%
IKDC score within 15th percentile of normal age– and sex-specific group point	53.8%
ACL-RSI score＞56	30.8%

Analysis for patients received hamstring tendon autograft

Significant differences between affected and contralateral limbs were observed in the quadriceps strength (*P* < 0.001), hamstring strength (*P* = 0.003), SH distance (*P* < 0.001), and TH distance (*P* < 0.001) at 9 months after ACLR. Affected limbs had smaller rotational angles than did contralateral limbs (*P* = 0.003); no significant difference in other kinematic parameters was observed. The mean kinematics LSIs did not exceed 80%, except for that for the flexion angle ROM (Supplementary Table 5).

Four (14.81%) patients fulfilled the criteria of the test protocol. Ten patients fulfilled the quadriceps and 11 patients fulfilled the hamstring strength criteria, 9 patients fulfilled the SH criterion, 11 patients fulfilled the TH criterion, and 22 patients fulfilled the knee laxity criterion. Twenty, 6, 6 patients fulfilled the flexion-extension, varus-valgus, and internal-external rotation ROM criterion. Five, 8, and 7 patients, respectively, fulfilled the anterior-posterior, proximal-distal, medial-lateral translation ROM criterion. Fourteen patients fulfilled the IKDC scale score criterion and 10 patients fulfilled the ACL-RSI scale score criterion (Supplementary Table 6).

The trend of the results and conclusion are consistent with the overall group.

Supplementary Table 4 Participant characteristics of patients received hamstring tendon autograft

	Patients
Sex: male/female, n	24/3
Injured knee: left/right, n	11/16
Age, y	27.7±6.0
Height, cm	178.7±6.5
Weight, kg	77.4±14.3
BMI, kg/m^2	24.1±3.4
Time post-surgery, m	9.3±0.5
IKDC score	81.6±11.1
ACL-RSI score	50.0±9.7

BMI: body mass index; IKDC: International Knee Documentation Committee Subjective Knee Evaluation Form; ACL-RSI: Anterior Cruciate Ligament-Return to Sport After Injury Scale

Supplementary Table 5 Functional and kinematics parameters [a]

	Affected limb	Contralateral limb	LSI	P value
Functional tests				
Extensor strength, Nm/kg	1.5 ± 0.5	1.9 ± 0.6	83.8 ± 19.4	< 0.001*
Flexor strength, Nm/kg	0.9 ± 0.3	1.1 ± 0.3	86.2 ± 25.2	0.003*
SLH, cm	127.9 ± 15.2	145.5 ± 13.3	87.9 ± 6.1	< 0.001*

TLH, cm	402.2 ± 63.5	460.6 ± 50.5	87.1 ± 7.8	< 0.001*
Laxity, mm	16.5 ± 3.0	15.8 ± 3.2		0.227
Knee kinematics				
F-E, °	57.4 ± 5.3	58.1 ± 4.9	91.8 ± 6.6	0.565
IR-ER, °	14.3 ± 3.6	16.8 ± 3.5	77.2 ± 12.5	0.003*
VR-VL, °	12.4 ± 4.6	13.8 ± 4.8	74.5 ± 16.3	0.145
A-P, mm	25.4 ± 7.9	27.6 ± 11.3	68.9 ± 16.1	0.353
P-D, mm	25.7 ± 9.7	24.6 ± 10.4	74.9 ± 23.2	0.585
M-L, mm	14.8 ± 5.2	14.2 ± 6.8	72.3 ± 16.7	0.589

[a] Data are expressed as mean ± standard deviation; F-E: flexion-extension; IR-ER: internal-external rotation; VR-VL: varus-valgus; A-P: anterior-posterior translation; M-L: medial-lateral translation; P-D: proximal-distal translation; *Statistically significant difference (P < 0.05)

Supplementary Table 6 Rates of criterion fulfillment

Pass criteria	Percentage of subjects passed criterion
LSI > 90 % peak torque quadriceps 60°/s	37.0%
LSI > 90 % peak torque hamstrings 60°/s	40.7%
LSI > 90% SLH test	33.3%
LSI > 90% TLH test	40.7%
Bilateral displacement difference of knee joint < 3mm	81.5%
Differences of ROM in flexion-extension < 10%	70.4%
Differences of ROM in varus-valgus < 10%	22.2%
Differences of ROM in internal-external rotation < 10%	22.2%
Differences of ROM in anterior-posterior translation < 10%	18.5%
Differences of ROM in proximal-distal translation < 10%	29.6%
Differences of ROM in medial-lateral translation < 10%	29.6%
IKDC score within 15th percentile of normal age– and sex-specific group point	51.9%
ACL-RSI score > 56	37.0%
